# Why Haven’t We Found an Effective Treatment for COVID-19?

**DOI:** 10.3389/fimmu.2021.644850

**Published:** 2021-03-31

**Authors:** Alexander James Spicer, Sirpa Jalkanen

**Affiliations:** ^1^ MDP Drug Discovery and Development, Institute of Biomedicine, Faculty of Medicine, University of Turku, Turku, Finland; ^2^ MediCity Research Laboratory and Institute of Biomedicine, University of Turku, Turku, Finland

**Keywords:** COVID-19, anti-virals, type I interferons (IFNs), corticosteroids, clinical trials

## Introduction

The COVID-19 pandemic continues to grip the world, with significant ramifications not only for public health but also for all major economies and industries. The scientific and medical communities, as well as citizens worldwide, await the return to normality, with the belief that an effective vaccine/therapy will be discovered.

The pandemic began over a year ago, giving enough time to acquire the first positive preliminary results with respect to treatment of patients with COVID-19. These results inspired hope (1, 2). However, optimism was short-lived because more recent evidence conflicts with the initial positive results, thereby delaying the return to normality. These contradictory results may be based, at least partially, on our ignorance of the biology of viral disease progression, natural immune responses to the virus, and the timing of treatment with experimental therapeutics (3, 4).

## Antivirals

Remdesivir was the first real hope as a treatment for COVID-19; indeed, multiple governments flocked to repurpose this antiviral drug as a potential treatment (2). At present, numerous trials are evaluating Remdesivir, although the first double-blind, randomized clinical trial concluded that the drug improved neither mortality nor viral clearance. The trial was prematurely terminated due to more adverse events in the active arm. Therefore, we have insufficient data and cannot draw clear conclusions about the efficacy of Remdesivir (2). Recently released results from the WHO’s Solidarity trial provide more evidence of the ineffectiveness of Remdesivir as a treatment for COVID-19 (5). Therefore, this antiviral is unlikely to be the “silver bullet” for COVID-19, despite the initial optimism.

Some anti-viral therapies such as favipiravir have been overlooked throughout the pandemic (6) whilst anti-virals such as hydroxychloroquine and chloroquine were favoured for their global availability, inexpensiveness and superior *in vivo* results (7). The use of both, hydroxychloroquine and chloroquine still remains controversial but they have shown positive signs akin to many repurposed assets. These have been reviewed extensively by Younis et al. who provide an overview including a potential therapeutic window (8).

Also antibodies have been designed to reduce the viral load and they are undergoing major trials worldwide. Convalescent plasma had initial enthusiasm due to the extensive history of its use in infectious diseases and good availability from voluntary donors. However, this initial optimism led to a disappointment with failure in PLACID trial (9). In contrast, anti-COVID-19 antibodies turned out to be effective, if given during the early phase of the disease: the patients receiving the antibody had less severe clinical symptoms and a significant drop in viral load compared to those treated with placebo (10). These results point towards the necessity for early treatment with antivirals or agents, which neutralize the virus (10). These new antibody-based therapies, however, will be ineffective in future pandemics caused by other viruses. Moreover, they are expensive to produce at scale.

## Corticosteroids

Outside of the WHO’s Solidarity trial, other major trials have looked at the use of steroids to treat COVID-19, despite conflicting and controversial evidence suggesting their ineffectiveness in multiple viral pandemics and cases of acute respiratory distress syndrome (ARDS) (11, 12). However, limited studies show a marked improvement in patients’ condition after treatment with systemic corticosteroids. A study by Villar et al., reported that dexamethasone reduces the duration of mechanical ventilation and improves overall mortality; however, the patients were naïve to treatment and were in intensive care units, suggesting that they were in a serious condition after having already mounted a natural response to infection (13).

The RECOVERY trial conducted during the COVID-19 pandemic supports the conclusions made by Villar et al., i.e., that systemic glucocorticoids are an effective therapy (1). Although it was a large-scale trial, RECOVERY is not without fault; the main criticism is that it is an open-label study that lacks data to allow evaluation, whether the treatment arms were balanced. Despite this, the conclusions of the RECOVERY trial are similar to those of Villar et al. (1, 13). Indeed, the RECOVERY trial showed that patients suffering from the most severe COVID-19 symptoms and were on respiratory support had the best responses to glucocorticoid treatment (1, 13, 14). However, as pointed out by RECOVERY, the same treatment is not beneficial, and possibly even harmful, during the early stages of the disease when a natural antiviral response is required.

The RECOVERY trial showed that a regime based on systemic glucocorticoids should be instigated 7 days post-symptom onset, when the inflammatory response to COVID-19 has begun to cause lung damage (1). A meta-analysis published by the WHO REACT force supports the effectiveness of glucocorticoid therapy (15), but the significance of the results disappeared (12) when the RECOVERY results were removed from the analysis, suggesting that the regime is useful only for a subpopulation of patients.

Recently the WHO Solidarity trial concluded that antiviral agents had little to no effect on hospitalized COVID-19 patients compared with local standards of care. As per the recommendation by the WHO, systemic glucocorticoids should be reserved for the most severe cases of COVID-19. Indeed, early use is detrimental to patient survival (16). The utmost importance of treatment timing was highlighted by the WHO itself, but later disregarded during the Solidarity trial, the results of which show lack of efficacy of any drugs including glucocorticoids. In each antiviral treatment arm of the Solidarity trial, mortality was not altered when patients receiving and not receiving steroids were compared. Thus, glucocorticoids were shown to be ineffective in the real world setting (5).

Li et al. conducted a pilot study to identify diagnostic markers of a therapeutic window for effective glucocorticoid treatment (17). The group found that the therapeutic window for corticosteroids opened when lactase dehydrogenase levels were less than two times the upper limit of the normal value and coupled with marked radiographic progression of lung inflammation. This finding could also explain the reason why Villar et al. found controversial yet positive results when using corticosteroids; they administered the steroids when the natural immune response was waning and the patients began to experience excessive inflammation (13, 17). The results of Li et al. also provide evidence that steroids are ineffective and detrimental during the early stages of viral invasion and immune activation (17).

## Harmful Interactions of the Drugs—Have They Been Ignored?

Serious concerns have been raised because glucocorticoids block type I interferon (IFN) signalling pathways (18). This has been shown both *in vitro* and in previous clinical trials in which contamination of IFN-β by glucocorticoids led to therapy failure when IFN-β was given intravenously (18–20). Timing of a particular treatment is likely to be crucial and should reflect the different phases of the disease, as well as the natural immune response. Treatment of patients with MERS, and the results of the MIRACLE Study, show that early treatment with type 1 IFNs is necessary to achieve positive results. Indeed, patients treated within 7 days of symptom onset derived a clear benefit (3). Outside of COVID-19 patients the early type 1 IFN response is necessary in both primates and bats to defend themselves against other viruses (6). The beneficial effects of type I IFNs are lost, if they are given to patients at the later stages of the disease when treatment strategies should be aimed to suppress the cytokine storm in the biphasic manner seen in nature (6).

This treatment strategy aligns with the endogenous response to invasion by a pathogen (19, 21), and is likely to benefit those patients who have not been able to mount a sufficiently strong type I IFN response themselves (22). IFN-β seems to be the most likely type of IFNs to show a therapeutic effect due to the rarity of autoantibodies against it and its natural anti-inflammatory properties. During the immune activation stage after COVID-19 infection, endogenous type I IFNs are produced by innate immune cells; this step of the immune response is essential to combat viral infections and inhibit viral replication (23). The inability to mount an effective type I IFN response to the initial assault correlates with greater severity of COVID-19 (22, 24).

Several studies have been undertaken in an attempt to better understand the IFN responses and the efficacy of type I IFNs in previous coronavirus pandemics (3, 25). *In vitro* and clinical studies make it clear that IFN-β is superior to IFN-α. The most crucial considerations concerning the use of type I IFNs are the route of administration (26, 27) and timing of the treatment (25). Arabi et al. showed that treatment with IFNβ-1β was only useful during the first 7 days after symptom onset, while Hung et al. showed a similar benefit within 5 days. These trials point to the benefit of early IFN treatment. Indeed, late intervention with IFN may be futile (3, 22, 25, 28, 29).

Some reports show that patients with severe COVID-19 mount a robust IFN response; therefore, severe disease is likely due to overproduction of cytokines, such as TNF-α and interleukins such as IL-1 and IL-6 which opens the therapeutic window to a generic immunological dampener such as corticosteroids (30). Indeed, the later stage of the disease provides a new window for use of focused inhibitors of IL-1 or IL-6 as the disease enters the complicating phase and cytokine storm begins (21). However, these interleukin inhibitors should be utilized in a targeted manner for specific populations. Stone et al. and Leisman et al. have discussed these extensively (31, 32) and point towards insufficient patient stratification as a reason for further inevitable failures (31).

Importantly, it should be noted that IL-6 inhibitors show significant efficacy in diseases, such as cytokine release syndrome where IL-6 levels are nearly 100-fold greater than those in patients with COVID-19 (32). Ongoing studies and completed trials are bound to fail because none of the inclusion criteria included increased levels of targeted interleukins (31).

Recently, optimal timing of interleukin inhibitor treatments for successful outcome has been suggested and this is expected to reveal better clinical results (33, 34). Whilst still providing mixed results, the REMAP-CAP trial is likely to have captured enough patients at this immunological cusp i.e., among those patients moving to mechanical support (35). This may show benefit, whereas other trials may have missed the crucial timing.

## Timing is the Key

Our difficulty in understanding patients’ immune responses with respect to disease progression and to treat them accordingly has already led to unnecessary deaths, and will continue to do so unless we take into account endogenous immunological cues (19). Understanding this and changing therapeutic schemes to fit these therapeutic windows may lead to more positive results in COVID-19 trials (21). It is unlikely that continuing on the present course will lead to a miraculous wave of positive results.

**Figure 1:**
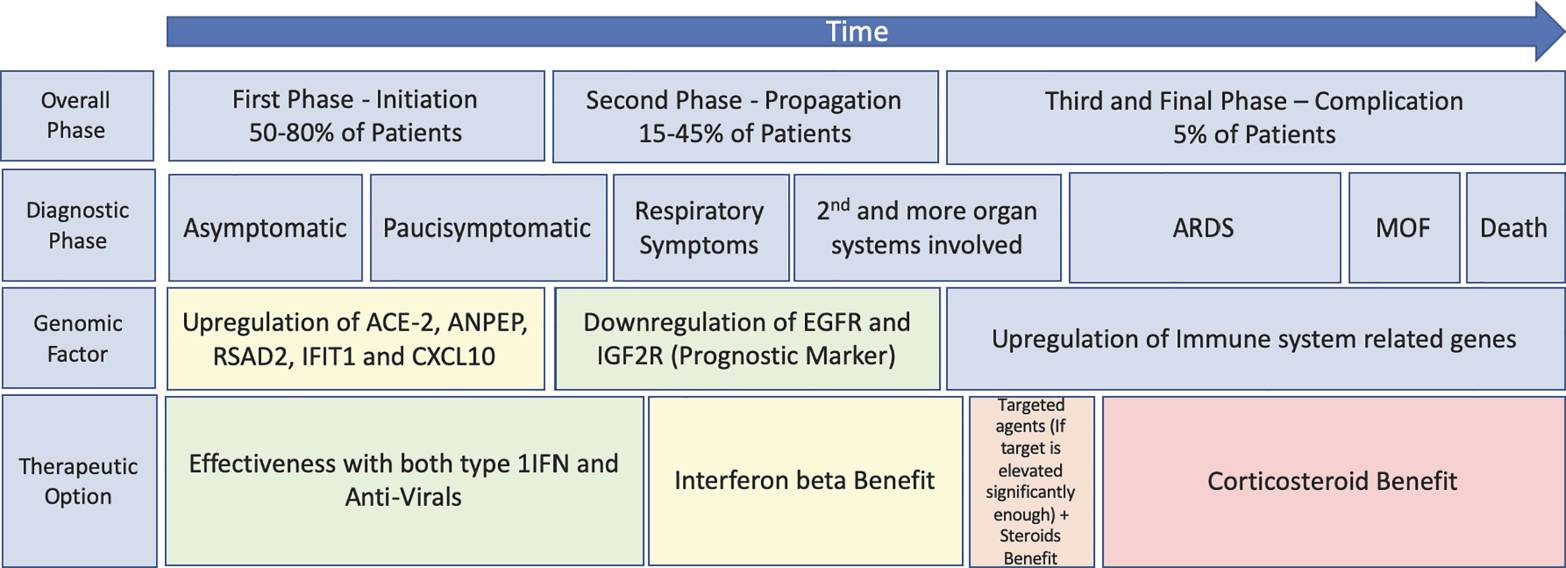
Schematic highlighting of the progression of SARS-CoV-2 and COVID using both Turk et al.’s three clinico-biological phases (21) and Feuillet et al.’s disease kinetic model for an outlook of the benefits of previously trialled therapeutics (6) (plotted against the disease course). MOF, multiple organ failure.

In conclusion, for any intervention against COVID-19, timing is crucial with the optimal therapeutics being administered in the early phases of disease manifestation, if we are to obtain the desired clinical response and prevent further deaths. Early therapeutic intervention will prevent patients entering the cytokine storm, alleviate pressure on ICUs and prevent further deaths (6). **Figure 1** shows a proposed therapeutic management regime for COVID-19, including the therapeutic windows for antiviral agents, IFNs, targeted therapies, and corticosteroids as well as prognostic and diagnostic markers allowing the clinicians provide the patients with a proper transition of therapeutics according to the different disease states. Thus, we believe that correct timing of these treatment options together with proper thromboprophylaxis (36) is expected to improve the outcome of the COVID-19 patients.

## Discussion

The idea that one therapeutic must be better than another is an adversarial jousting competition in which there can only be one winner; this is a dangerous path to tread. During the last two decades, a wealth of conflicting evidence has emerged from a variety of trials aimed at finding an effective treatment for coronavirus pandemics. This is even more pressing in the era of COVID-19.

During this era of “trialism”, basic pharmacology and biology have been forgotten in the search for a desperately needed therapeutic. Low quality, duplicated, prematurely stopped, and underpowered trials have been conducted, all of which have provided only limited understanding and perspectives on how to respond to and treat COVID-19. Resources should be redirected towards a high-quality, coordinated approach that unites basic science, pharmacology, clinical practice/evidence, and experienced trialists.

The battle lines were drawn early in the current pandemic, and doctrine biology was thrown out in the rush to bring a therapeutic to the market as quickly as possible. Conflicting results were generated unnecessarily. This could have been avoided by better understanding the nuanced processes in our own bodies. These controlled processes have had millions of years to develop and they defend us in the most optimal way. It would be unwise to forget this once again and continue on a “hunger games” path to drug development, which will lead to more people losing their lives to the virus. We need to understand the presented evidence and utilize it to develop treatments that provide effective results.

## Author Contributions

Both authors significantly contributed to this Opinion. All authors contributed to the article and approved the submitted version.

## Conflict of Interest

SJ owns stocks of Faron Pharmaceuticals.

The remaining author declares that the research was conducted in the absence of any commercial or financial relationships that could be construed as a potential conflict of interest.
